# Fear of Childbirth After Major Orthopedic Traumas: A Nationwide Multi‐Register Analysis

**DOI:** 10.1111/birt.12869

**Published:** 2024-08-30

**Authors:** Matias Vaajala, Rasmus Liukkonen, Ilari Kuitunen, Ville Ponkilainen, Ville M. Mattila, Maiju Kekki

**Affiliations:** ^1^ Faculty of Medicine and Life Sciences University of Tampere Tampere Finland; ^2^ Department of Pediatrics Mikkeli Central Hospital Mikkeli Finland; ^3^ Institute of Clinical Medicine and Department of Pediatrics University of Eastern Finland Kuopio Finland; ^4^ Department of Surgery Central Finland Central Hospital Nova Jyväskylä Finland; ^5^ Department of Orthopaedics and Traumatology Tampere University Hospital Tampere Finland; ^6^ Department of Obstetrics and Gynecology Tampere University Hospital Tampere Finland; ^7^ Center for Child, Adolescent and Maternal Health Research, Faculty of Medicine and Health Technology Tampere University Tampere Finland

**Keywords:** cesarean birth, epidemiology, fear of childbirth, trauma

## Abstract

**Background:**

The aim of this study was to evaluate the association between previous major traumas and the prevalence of fear of childbirth (FOC) and the subsequent effects of FOC on the intended mode of delivery.

**Methods:**

In this nationwide retrospective register‐based cohort study, data from the Care Register for Health Care were linked with the National Medical Birth Register (MBR) to evaluate the prevalence of FOC after major traumas. A total of 18,573 pregnancies met the inclusion criteria. A multivariable logistic regression model was used to assess the effects of FOC on the intended mode of delivery. Women with major traumas before pregnancy were compared to individuals with wrist fractures. Adjusted odds ratios (aORs) with 95% CIs between the groups were compared.

**Results:**

Of those pregnancies that occurred after major traumas, 785 (6.2%) women were diagnosed with FOC after traumatic brain injury (TBI), 111 (6.1%) women after spine fracture, 38 (5.0%) women after pelvic fracture, 22 (3.2%) women after hip or thigh fracture, and 399 (5.2%) women in the control group. Among those women diagnosed with FOC, the adjusted odds for elective CB as an intended mode of delivery were highest among women with previous spine fractures (aOR 2.28, CI 1.45–3.60) when compared to the control group.

**Conclusions:**

We found no evidence of differences in maternal FOC in patients with preceding major traumas when compared to the control group. Therefore, it seems highly likely that the major trauma itself is the explanatory factor for the increased rate of elective CB.

## Graphical abstract

The incidence of fear of childbrith after major orthopedic traumas.

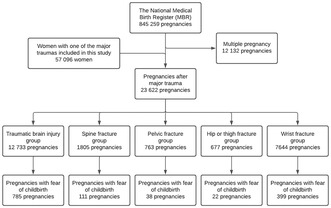

## 1 Introduction

Fear of childbirth (FOC) is a common obstetrical challenge affecting the health of women [1]. In addition to previous operative deliveries (vacuum or emergency cesarean delivery), higher socioeconomic status, advanced maternal age, and depression are all predictive factors for FOC [2, 3]. Moreover, FOC is a common reason for women to request a cesarean birth (CB) [4, 5, 6, 7]. In addition, women with FOC are known to have a higher need for labor analgesia and a lower birth rate in the Finnish population [8, 9].

Higher rates of CBs have previously been observed among women who sustained a major trauma, such as traumatic brain injury (TBI) or fractures of the spine or pelvis, before becoming pregnant [10, 11]. Indeed, the rates for CB are reported to rise to 19.2% after TBI, 19.7% after spine fracture and 22.6% after pelvic fractures. This increase is remarkable when compared to the overall CB rate of approximately 16% in Finland during the last decades [12]. Moreover, a possible lower total birth rate has also been observed after previous major traumas. In addition, the rates of CB among these women remain high even after a long follow‐up [13, 14].

As FOC is associated with an increased risk for CB, it is necessary to evaluate how preceding major traumas affect both the risk for FOC and the number of Cesareans performed. We hypothesized that the prevalence of FOC might increase after major traumas, such as pelvic fractures and spine fractures, that are in the area of the reproductive system and, therefore, cause uncertainty in the minds of women over their capacity to give birth vaginally. In this study, we aim to evaluate the effects of major traumas on the prevalence of FOC and the subsequent effects of FOC on the intended mode of delivery.

## 2 Materials and Methods

In this nationwide retrospective register‐based cohort study, data from the Care Register for Health Care were linked with the National Medical Birth Register (MBR) to evaluate the prevalence of FOC after major traumas. The study period was from 1 January 2004 to 31 December 2018.

The Care Register for Health Care contains data on patients discharged from inpatient care, the number of patients in inpatient care in health centers and hospitals on 31 December, day surgeries, and specialized outpatient care. The coverage and quality of the register are high. Thirteen International Classification of Diseases 10th revision codes (ICD‐10) found in the Care Register for Health Care were used to identify the specific trauma patients experienced. Traumatic brain injuries (TBIs), spine fractures, pelvic fractures, and hip or thigh fractures were included in the study. Women with wrist fractures were chosen as a control group because these are generally minor traumas and are located in the distal limb far from the reproductive system. They are not, therefore, believed to arouse major fears or uncertainties about pregnancy and childbirth. In addition, as a trauma population, women with wrist fractures are believed to better represent the backgrounds and behaviors of the general population and thus make a viable reference group. The specific ICD‐10 codes with definitions for each trauma included in this study are presented in Table S1.

The MBR is maintained by the Finnish Institute for Health and Welfare (THL) and contains data on pregnancies, delivery statistics, and the perinatal outcomes for all births with a birthweight of ≥500 g or a gestational age of ≥22 + 0 weeks. The MBR has high coverage and quality (the current coverage is nearly 100%) [15, 16]. In Finland, all pregnant women are asked about any fears they may have about childbirth during antenatal visits. Women experiencing a significant FOC who cannot be helped during the antenatal visits to women and child welfare clinics and/or those who have made a CB request due to FOC are referred to maternity clinics. If a woman is referred to maternity care for this reason or if the FOC manifests some time over the course of care, the woman is cared for by physicians or specialized midwives during maternity care visits. In the present study, FOC was defined according to the ICD‐10 code O99.80, which was first established in 1997. There are, however, no uniform criteria or definitions for FOC. In this study, FOC is defined as anxiety and fear of pregnancy, childbirth, or parenting of a child that impair daily wellbeing, which is recognized by physicians' subjective evaluation during visits in maternity care. However, FOC takes different forms in different women, and may also manifest as physical complaints, nightmares, and difficulties in concentrating [17]. The diagnosis of FOC has been registered in the MBR since 2004.

**FIGURE 1:**
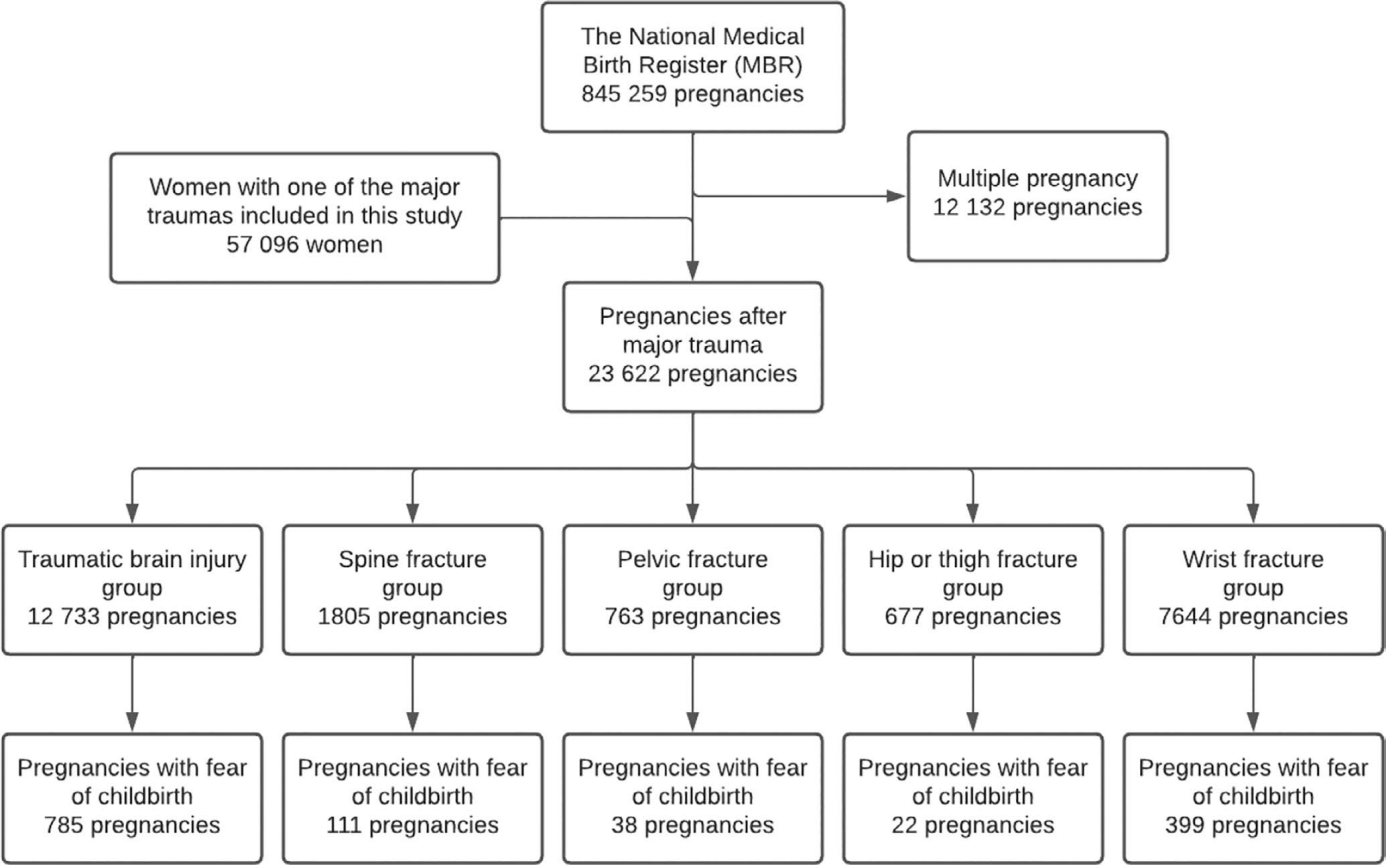
Flowchart of the study populations. Data from the MBR were combined with data on the diagnosed major trauma hospitalizations in the Care Register for Health Care.

All singleton pregnancies that occurred after one of the traumas described above were included in this study. In total, data on 17,629 women who had sustained one of the traumas were collected from the Care Register for Health Care. The date of the fracture and the date of the beginning of pregnancy were used to identify all singleton pregnancies occurring after different traumas. A total of 18,573 pregnancies met the inclusion criteria. The process used to form the study groups is presented as a flowchart in Figure 1.

## 3 Analysis

Continuous variables were interpreted as means with standard deviations (SDs) or as a median with an interquartile range (IQR) based on the distribution of the data. The categorical variables are presented as absolute numbers and percentages. Student's *t*‐test, Mann–Whitney *U*‐test, and Chi‐Squared tests were used for group comparisons. *p*‐Value under 0.05 was considered statistically significant. The multivariable logistic regression model was used to assess the effects of FOC on the intended mode of delivery. Women with major traumas before pregnancy were compared to a control group comprising pregnancies after wrist fractures. The exposure variable was the type of major trauma. The outcome was the intended mode of delivery (elective CS/trial of labour). Adjusted odds ratios (aORs) with 95% CIs between the groups were compared. The analyses were stratified based on the diagnosed FOC. The model was adjusted by maternal age, previous cesarean section, and weight of the neonate because the increasing age of the mother, increasing size of the neonate, and previous CS are all known to be indications for elective CS [18, 19, 20]. Adjustments were made by choosing the variables for a multivariable model using directed acyclic graphs (DAGs) constructed using the free online software DAGitty (dagitty.net). The variables included in the DAGs were chosen based on known risk factors and hypothesized causal pathways. Based on previous literature, the known risk factors for elective CS are previous CS, increased maternal age, and higher neonatal weight [20, 21, 22, 23]. DAGitty automatically suggests possible adjustment variable sets that can influence the main outcome. DAG is shown as Figure S1. The results of this study are reported according to STROBE guidelines [24]. Statistical analyses were performed using R version 4.0.3 for Windows (R Foundation for Statistical Computing, Vienna, Austria).

## 4 Results

Of those pregnancies occurring after major traumas, a total of 785 (6.2%) women were diagnosed with FOC after TBI, 111 (6.1%) after spine fracture, 38 (5.0%) after pelvic fracture, 22 (3.2%) after hip or thigh fracture and 399 (5.2%) after wrist fracture in the control group (*p* < 0.01). Women in the major trauma groups were younger at the time of pregnancy when compared to the reference control group (*p* < 0.01). Women in the pelvic fracture group had the highest rate of elective CB (11.4%) as the intended mode of delivery, followed by the spine fracture group (9.1%), the TBI group (7.6%), the hip or thigh fracture group (7.1%) and the control group (5.8%) (*p* < 0.01). (Table 1) Of those women who were diagnosed with maternal FOC in each trauma group, 274 (34.9%) had elective CBs in the TBI group, 60 (54.1%) in the spine fracture group, 16 (42.1%) in the pelvic fracture group, 7 (31.8%) in the hip or thigh fracture group and 148 (37.1%) in the control group (*p* < 0.01).

**TABLE 1:** Background information on the study groups.

	TBI group		Spine fracture group		Pelvic fracture group		Hip or thigh fracture group		Control group	
	*n*	%	*n*	%	*n*	%	*n*	%	*n*	%
Total number of pregnancies	12,733		1805		763		677		7644	
Age (mean; SD)	28.7 (5.4)		29.5 (5.2)		29.2 (5.2)		29.0 (5.5)		30.4 (5.3)	
Smoking status										
Smoker	3513	27.6	483	26.8	176	23.1	163	24.1	1484	19.4
Nulliparous	5601	44.0	840	46.5	331	43.4	315	46.5	3418	44.7
Previous CS	1501	11.8	184	10.2	91	11.9	61	9.0	832	10.9
Neonatal length, cm (mean; SD)	49.9 (2.5)		49.9 (2.6)		50.0 (2.5)		49.9 (2.7)		50.1 (2.4)	
Neonatal weight, g (mean; SD)	3487 (555)		3481 (561)		3466 (539)		3487 (568)		3517 (540)	
Intended mode of delivery										
Trial of labor	11,759	92.4	1640	90.9	676	88.6	629	92.9	7100	92.9
Elective CS	974	7.6	165	9.1	87	11.4	48	7.1	544	7.1
Diagnosed maternal fear of labor	785	6.2	111	6.1	38	5.0	22	3.2	399	5.2

Abbreviation: CS, cesarean section.

Among women with diagnosed FOC, the adjusted odds for elective CB as the intended mode of delivery were highest among those women with previous spine fractures (aOR 2.28, CI 1.45–3.60) when compared to the control group. However, we did not find any evidence of a difference in odds for elective CB as an intended mode of delivery for women with previous TBI, pelvic fracture, or hip or thigh fracture among women diagnosed with FOC. Among those women with no FOC, the odds for elective CB after pelvic fracture were higher (aOR 2.06, CI 1.53–2.73) when compared to the control group. We found no evidence of any difference in odds for elective CB as an intended mode of delivery for women with previous TBI, spine fracture, or hip or thigh fracture. (Table 2).

**TABLE 2:** Adjusted odds ratios (aOR) with 95% confidence intervals (CI) in the event of turning mode of delivery into elective cesarean section.

	Trial of labor		Elective CS		aOR CI
	*n*	%	*n*	%	
Trauma					
Traumatic brain injury					
FOC	514	65.4	271	34.5	0.92 (0.71–1.21)
Non‐FOC	11,177	94.1	692	5.9	1.10 (0.95–1.26)
Spine fracture					
FOC	51	45.9	60	54.1	2.28 (1.45–3.60)
Non‐FOC	1578	93.8	104	6.2	1.26 (0.98–1.59)
Pelvic fracture					
FOC	22	57.9	16	42.1	1.45 (0.70–2.97)
Non‐FOC	651	90.2	71	9.8	2.06 (1.53–2.73)
Hip or thigh fracture					
FOC	15	68.2	7	31.8	0.90 (0.32–2.32)
Non‐FOC	610	93.7	41	6.3	1.21 (0.83–1.73)
Control group					
FOC	251	62.9	148	37.1	—
Non‐FOC	6848	94.5	396	5.5	—

*Note:* The analyses were stratified based on the diagnosed fear of childbirth (FOC) and then the model was adjusted by maternal age, previous cesarean section, and length and weight of the neonate, as these factors are known to increase the odds for CS. Major trauma groups were compared to control group.

## 5 Discussion

We did not find any difference in maternal FOC in women who had sustained major traumas when compared to women with wrist fractures in the control group. Also, despite the lower or similar rates of FOC among women with previous pelvic or spine fractures, the odds for elective CB were markedly higher in these groups.

A high prevalence of FOC has recently been reported in the literature. Indeed, the total prevalence of FOC reported in a large study from China was nearly 68% [25]. In one multicenter study conducted in Ireland, the prevalence of severe FOC was 5.3%, and FOC was 36.7%. In our study, the prevalence of FOC increased from 1.1% to 3.6% in nulliparous women and from 1.5% to 7.8% in multiparous women. Despite these findings, the prevalence of FOC among women with a history of major trauma was similar to that among women in the control group who sustained a wrist fracture. Interestingly, the rate of FOC after major traumas near the reproductive system, such as pelvic fractures and hip or thigh fractures, was not higher than in the control group. It appears that previous adverse birth events have a stronger effect on the development of FOC than previous major traumas, as in a recent study, women with different previous birth complications had markedly higher risk for the development of FOC [26].

The odds of requesting an elective CB after pelvic fractures were higher even after taking the diagnosed maternal FOC into account. Based on the previous literature, the overall rate for CB was notably high after pelvic fractures in Finland, which was further explained by the increased rates of elective CB [27]. Another study that examined the effects of the time difference between pelvic and hip fractures revealed that the rate of elective CB after pelvic fracture remained high even after long‐term follow‐up [14]. These studies concluded that vaginal delivery after pelvic fracture was possible in the majority of cases and questioned the necessity of the high rates of elective CB reported after these major traumas [14, 27]. The results of this study support these concerns, as it appears that the maternal fear of giving birth is not more common after pelvic fractures. Furthermore, despite the increased rates of CB after pelvic fractures, the Cesarean rates in Finland have remained relatively low. In a previous systematic review of level 1 trauma centers, the rate of elective CB was reported to be over 40% after pelvic fractures [28]. Moreover, the rates of fear of labor in this group were not markedly higher when compared to the women with wrist fractures in the control group. Therefore, it seems that the major orthopedic trauma itself is most likely the explanatory factor for the increased rate of elective CB, which might, in turn, be caused by a lack of information and uncertainties in the minds of women about their capacity to give birth vaginally after major traumas. Interestingly, women who had sustained a previous TBI had the highest rate of FOC. According to a recent study, the rate of elective CB was also higher after TBI in the Finnish population [11]. However, the precise reason for this finding remains unknown. FOC may one part of the explanation, but the etiology of FOC after TBI remains unknown. One explanation for the higher rate of elective CB after major traumas might be the underlying psychological factors affecting the intended mode of delivery. This may be especially so among individuals with TBIs, though other traumas are also known to have effects on psychological well‐being, increasing the risk for mental health disorders, including depression, post‐traumatic stress disorder, and personality disorders [29, 30]. Psychological challenges are important factors, as many of these, such as lower self‐esteem, greater perceived stress, and depression are known to be related to elective CB as an intended mode of delivery [31, 32].

CB is linked to a decrease in the mortality of neonates and parturients in selected cases. However, the drawbacks of CB for the neonate include increased risk for asthma, obesity, and poorer cardiorespiratory health in later life as compared to those born vaginally [33, 34]. Additionally, breastfeeding duration is shorter after elective CB [35]. For women, CB may cause pregnancy‐related complications in future pregnancies, and a higher risk for postoperative complications, such as pain, endometritis, wound separation/infection, urinary tract infection, gastrointestinal problems, deep venous thrombosis, and septic thrombophlebitis [36, 37]. It is, therefore, important to study the indications behind the high rate of elective CB and the necessity of this procedure after different major traumas. As our results clearly show, the prevalence of FOC is not markedly higher after major traumas, and the notably increased rates of elective CB might therefore be explained by a lack of knowledge on the part of the clinician regarding the capability of women to give birth vaginally after major traumas, as the literature on this topic is limited to only a few studies [10, 14, 27]. Taken together, the results of this study and the existing literature should encourage clinicians and patients who have sustained previous major traumas to consider the possibility of vaginal delivery.

The strengths of our study are the large nationwide register data used and the long study period, which allowed us to analyze the rates of FOC using a large study population. The register data used in our study are routinely collected in structured forms using national instructions, which ensures good coverage (over 99%) and reduces possible reporting and selection biases. The main limitation of this study is that the indications for elective CB are not registered in the MBR, which means the reasons for this delivery method remain unknown. In addition, there is no uniform criteria or definition for FOC. Moreover, we cannot rule out variations in diagnosing practices which may have led to the underreporting of the O99.80 diagnosis among women with major trauma. In addition, FOC takes different forms in different women, often manifesting as physical complaints, nightmares, and difficulties in concentrating [17], making it challenging to diagnose. Also, the severity of traumas remains unknown because the traumas are only based on the registered ICD‐10 code found in the Care Register for Health Care. In addition, some women in the trial of labor group with a recorded urgent CB may already have planned an elective CB, but because the labor began early, the planned elective CB was recorded as an urgent CB and, as a result, was misinterpreted as an attempted vaginal delivery. Also, we have no information on psychological challenges, other comorbidities, or other complications (e.g., complications in previous pregnancies) among women with major traumas, that might have an effect on the intended mode of delivery.

## 6 Conclusion

We did not find any evidence of a difference in maternal FOC in patients with major traumas when compared to the control group. Therefore, the major orthopedic trauma itself is most likely the explanatory factor for the increased rate of elective CB. These results should be acknowledged by the clinician when the mode of delivery is discussed with the patient.

## Conflict of Interest

The authors declare no conflicts of interest.

## Supporting information


Figure S1

Table S1

## Data Availability

The data that support the findings of this study are available from Findata. Restrictions apply to the availability of these data, which were used under license for this study. Data are available from the author(s) with the permission of Findata.
