# Supplementary material for: Fear of Childbirth After Major Orthopedic Traumas: A Nationwide Multi‐Register Analysis
Source: Birth. 2024 Aug 30;52(1):123–8. doi: 10.1111/birt.12869 (PMC11829265; doi:10.1111/birt.12869)
Supplement: Supplementary file 1 — Figure S1 [file BIRT-52-123-s001.pdf]

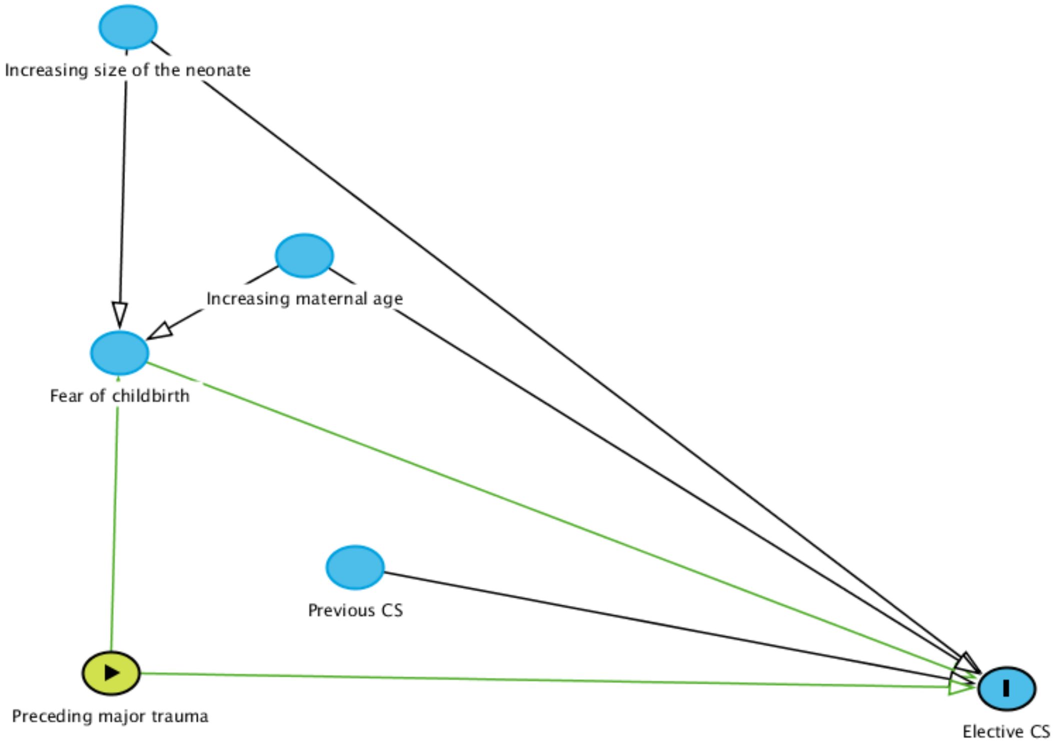


Supplementary figure 1: DAG: Major trauma as an exposure variable, and elective cesarean section (CS) as an outcome.
