# Supplementary material for: Fear of Childbirth After Major Orthopedic Traumas: A Nationwide Multi‐Register Analysis
Source: Birth. 2024 Aug 30;52(1):123–8. doi: 10.1111/birt.12869 (PMC11829265; doi:10.1111/birt.12869)
Supplement: Supplementary file 2 — Table S1 [file BIRT-52-123-s002.pdf]

Supplementary table 1. ICD-10 (International Classification of Diseases 10th revision) codes with definitions for each major trauma group and reference group included in this study.

TBI	
ICD-10 code	Definition
S06.0	Concussion
S06.1	Traumatic cerebral edema
S06.2	Diffuse traumatic brain injury
S06.3	Focal traumatic brain injury
S06.4	Epidural hemorrhage
S06.5	Traumatic subdural hemorrhage
S06.6	Traumatic subarachnoid hemorrhage
S06.8	Other specified intracranial injuries
S06.9	Unspecified intracranial injury
Spine traumas	
ICD-10 code	Definition
S12.0	Fracture of first cervical vertebra
S12.1	Fracture of second cervical vertebra
S12.2	Fracture of third cervical vertebra
S12.7	Multiple fractures of cervical vertebra
S12.8	Fracture of other parts of neck
S12.9	Fracture of neck, unspecified
S22.0	Fracture of thoracic vertebra
S22.1	Multiple fractures of thoracic vertebra
S32.0	Fracture of lumbar vertebra
Pelvic traumas	
ICD-10 code	Definition
S32.1	Fracture of sacrum
S32.3	Fracture of ilium
S32.4	Fracture of acetabulum
S32.5	Fracture of pubis
S32.7	Multiple fractures of lumbar spine and pelvis
S32.8	Fracture of other parts of pelvis
S32.9	Fracture of unspecified parts of lumbosacral spine and pelvis
Hip or thigh traumas	
ICD-10 code	Definition
S72.0	Fracture of head and neck of femur
S72.1	Pertrochanteric fracture
S72.3	Fracture of shaft of femur
S72.4	Fracture of lower end of femur
S72.7	Multiple fractures of femur
S72.8	Other fracture of femur
S72.9	Unspecified fracture of femur
Wrist traumas	

ICD-10 code	Definition
S52.5	Fracture of distal end of radius
S52.6	Fracture of distal end of ulna
S62.0	Fracture of navicular bone of wrist
S62.1	Fracture of other and unspecified carpal bone
S62.2	Fracture of first metacarpal bone
S62.3	Fracture of other and unspecified metacarpal bone
S62.4	Multiple fractures of metacarpi
